# Intra-generational protein malnutrition impairs temporal astrogenesis in rat brain

**DOI:** 10.1242/bio.023432

**Published:** 2017-05-25

**Authors:** Aijaz Ahmad Naik, Nisha Patro, Pankaj Seth, Ishan K. Patro

**Affiliations:** 1 School of Studies in Neuroscience, Jiwaji University, Gwalior 474011, India; 2 School of Studies in Zoology, Jiwaji University, Gwalior 474011, India; 3 National Brain Research Centre, Manesar, Haryana 122051, India

**Keywords:** Astrocyte, GFAP, BLBP, Protein malnutrition, Hippocampus, S100β

## Abstract

The lack of information on astrogenesis following stressor effect, notwithstanding the imperative roles of astroglia in normal physiology and pathophysiology, incited us to assess temporal astrogenesis and astrocyte density in an intra-generational protein malnutrition (PMN) rat model. Standard immunohistochemical procedures for glial lineage markers and their intensity measurements, and qRT-PCR studies, were performed to reveal the spatio-temporal origin and density of astrocytes. Reduced A_2_B_5_+ glia restricted precursor population in ventricles and caused poor dissemination to cortex at embryonic days (E)11-14, and low BLBP+ secondary radial glia in the subventricular zone (SVZ) of E16 low protein (LP) brains reflect compromised progenitor pooling. Contrary to large-sized BLBP+ gliospheres in high protein (HP) brains at E16, small gliospheres and discrete BLBP+ cells in LP brains evidence loss of colonization and low proliferative potential. Delayed emergence of GFAP expression, precocious astrocyte maturation and significantly reduced astrocyte number suggest impaired temporal and compromised astrogenesis within LP-F1 brains. Our findings of protein deprivation induced impairments in temporal astrogenesis, compromised density and astrocytic dysfunction, strengthen the hypothesis of astrocytes as possible drivers of neurodevelopmental disorders. This study may increase our understanding of stressor-associated brain development, opening up windows for effective therapeutic interventions against debilitating neurodevelopmental disorders.

## Abstract (precis)

**Summary:** Maternal protein deprivation results in low progenitor pooling, and delayed and compromised astrogenesis, suggesting astrocyte impairment as a driver of neurological diseases owing to their imperative roles in normal and pathological situations.

## INTRODUCTION

Brain development is a complex process with a specified timeline involving a series of successive and overlapping events, namely: (i) progenitor pooling and proliferation of embryonic stem cells; (ii) neurogenic phase and formation of cortical neurons; (iii) gliogenesis; (iv) myelination; (v) axon pruning, synaptic stabilization and apoptosis (Rowitch and Kriegstein, 2010; Jiang and Nardelli, 2016). These processes occur along specific timelines also known as temporal benchmarks or critical windows, i.e. an interval during development when the generation and formation of specific cell types and/or associated circuits develops (Semple et al., 2013). The developing brain is highly susceptible to insults at these critical windows leading to severe structural and functional impairments during later life in survivors (Andersen, 2003; Hensch, 2004; Patro et al., 2009, 2011). The privileged nature of the neurons places them on the first order in the developmental plan, followed by the astrocytes and oligodendrocytes. Neurogenesis in the cortical and subcortical structures commences in rodents around embryonic day (E)10.5 and is completed by postnatal day (P)15 (Bayer et al., 1992; Rice and Barone, 2000; Patro et al., 2015). Following the peak neurogenesis at E14, there is a shift from neurogenesis to astrogenesis where the astrocytes are generated either directly from primary radial glia (RGs) and glia restricted precursors (GRPs), from secondary RGs of subventricular zone (SVZ), or through the local proliferation of newly born immature astrocytes (Pinto and Götz, 2007; Ge et al., 2012; Molofsky and Deneen, 2015).

Astrogenesis, an important aspect of neural development, is critical for the normal physiology, cytoarchitecture and neuronal functioning. Glia account for almost 75% of cells within the brain and are implicated in a myriad of neurodegenerative and neuropsychiatric disorders including Alexander's disease, schizophrenia, depression, alcoholism, and suicide (Bernstein et al., 2009; Hercher et al., 2009; Torres-Platas et al., 2011; Sloan and Barres, 2014). Astroglia encompass a myriad of morphological entities that co-exist, and are characterized by unique origin, particular molecular signature, and specific spatial localization and function within the central nervous system (CNS). The existence of two major subtypes of astrocytes, the fibrous and the protoplasmic, is well established based on both the morphological differences and spatial location; however, recent studies also support the differences in origin (Tabata, 2015). Astrocytes are the most abundant type of glia in brain that outnumber neurons and play vital roles in energy metabolism, K+ ion buffering and neurotransmitter recycling, blood brain barrier (BBB) formation, and maintenance of neural circuits by controlling synapse formation, elimination and maturation (Clarke and Barres, 2013; Chung et al., 2013; Bayraktar et al., 2015). Astrocytic dysfunction can result in developmental and neuropsychiatric disorders and associated pathophysiology (Molofsky et al., 2012; Sloan and Barres, 2014).

The astrocyte precursor cells are derived from various progenitor populations like the neural stem cells (NSCs), RGs, and immature astrocytic precursor cells (APCs). The continuum of this differentiation process detection is based on the expression of specific molecular markers for every stage. A_2_B_5_, BLBP, Nestin and S100β are the reliable markers for the precursors. The others, like GFAP and S100β, correlate with the differentiation along astrocyte lineage and maturation. S100β is expressed in embryonic radial glia of both the ventricular and sub-ventricular zones and postnatally developing cerebella and has been reported as a potent marker of the radial glia together with other markers like BLBP, SOX-9, RC1 and RC2 (Hartfuss et al., 2001; Patro et al., 2015; Tabata, 2015). However, the re-expression of S100β postnatally has been accepted to be a marker for astrocytic maturation (Garcia et al., 2004; Raponi et al., 2007; Patro et al., 2015).

The generation of the appropriate number of astrocytes along the specific spatio-temporal timeline is a prerequisite for normal CNS cytoarchitecture, circuit organization and information processing in CNS. Therefore, any defects in the mechanisms involved in astrogenesis and their maturation during foetal and early postnatal life may be potential causative factors in the origin and propagation of many neurodevelopmental disorders. The normal cytoarchitecture and physiological development of the brain and consequent behaviour evolves from continuous interactions of the environmental and genetic components. Amongst all other environmental stressors, protein malnutrition (PMN) is a major variable known to be affecting the developmental plan and increasing risk for late onset disorders as reviewed in Alamy and Bengalloun (2012). Maternal PMN in particular is extremely detrimental for normal CNS development and has been extensively researched in animal models, found to alter neuronal populations, migration, myelination, synaptogenesis, hippocampal formation and neuronal transmission (Alamy and Bengelloun, 2012; Nyaradi et al., 2013). Information, however, on gliogenesis and associated changes following PMN is lacking.

As the timing of the appearance of various cell types during CNS development is orchestrated on a precise schedule and is critical for the normal cytoarchitecture (Kohwi and Doe, 2013), any insult may impair the timing and population of cells and ultimately drastically disrupt the normal developmental plan. The past decade of research has witnessed remarkable roles of astrocytes and other glial cells in neurodegenerative and neuropsychiatric disorders forcing for a revaluation of their traditional roles (Clarke and Barres, 2013; Verkhratsky et al., 2014; Oliveira et al., 2015; Tewari and Seth, 2015). These studies incited us to propose the hypothesis: does glial pathology precede neuronal pathology? Does protein insufficiency in maternal blood affect the normal gliogenesis cycles, and what type of compromise would the developing brains present?

Surprisingly enough, to our knowledge, no previous study has addressed gliogenesis in relation to maternal PMN, even now we have understood the importance of glial cells in neuronal development, survival and plasticity. Thus, the present investigation is focussed on understanding how maternal PMN would influence the temporal astrogenesis, density/turnover and subsequent mental health at adulthood and senility. Taken together, this study provides novel information on the effects of maternal PMN on astrogenesis that may open up windows for effective therapeutic clinical applications.

## RESULTS

### Discrete A_2_B_5_+ GRPs populate the ventricles during early embryogenesis and subsequently migrate to cortex

**Fig. 1.**
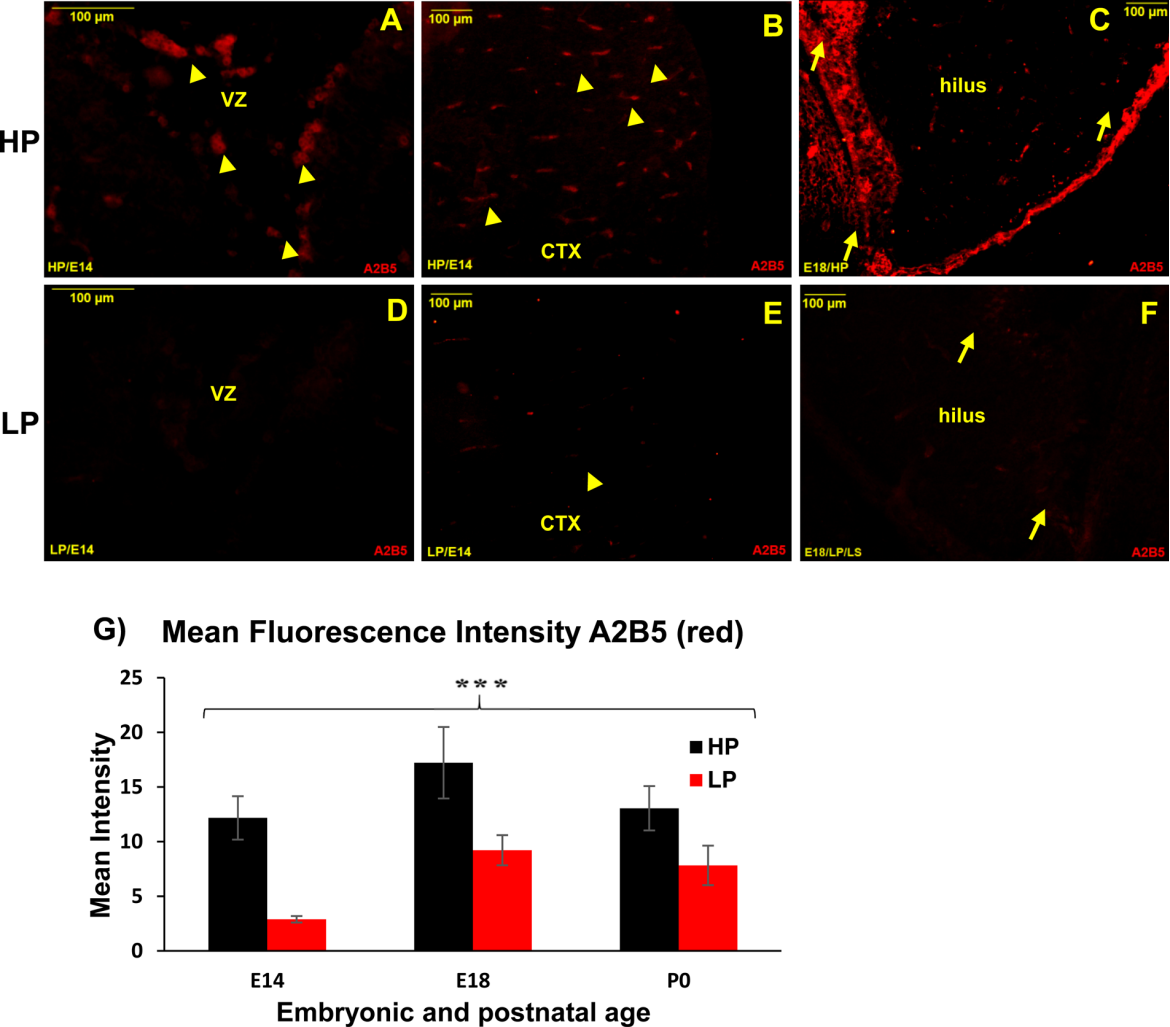
**Reduced A_2_B_5_+ GRP population in embryonic brains following maternal PMN.** Immunofluorescence labelling of sagittal sections through the developing forebrain with anti-A_2_B_5_ antibody evidence drastically low A_2_B_5_+ GRP population in the LP E14 ventricles (VZ, D) as compared to strongly immunopositive A_2_B_5_+ GRP clusters in HP counterparts (yellow arrowheads; A). Also, an appreciable number of anti-A_2_B_5_+ GRPs (yellow arrowheads) were seen migrating to populate cortex (CTX) of E14 HP brains (B) and clusters of A2B5+ GRPs (arrows) in hilus region at E18 (C) as compared to a very few weakly labelled GRPs (arrowhead; E) in the LP E14 cortex and E18 hilus (arrows; F). Scale bars: 100 µm. (G) Histogram shows relative changes as mean fluorescence intensity of A_2_B_5_ (red) immunoreactivity in LP versus HP sections measured with Fiji ImageJ software (NIH) evidence significantly decreased A_2_B_5_ expression in LP group. Error bars indicate s.e.m.; ***P≤0.025-0.001 (Student's *t*-test).

GRPs were selectively localized by A_2_B_5_ immunoreactivity in embryonic brains and found to be either clustered in ventricles or seen migrating to populate the cortex from E12-16. The ventricular clusters and singular bipolar A_2_B_5_+ GRPs were very prominently noticed in the high protein (HP) group preparations at E14 (Fig. 1A). In addition, the A_2_B_5_+ GRPs were also noticed in some non-ventricular areas, like pre-optic, lateral and medial ganglionic eminence of HP brains. However, such ventricular clusters of A_2_B_5_+ GRPs were not at all seen in the E14 low protein (LP) brains (Fig. 1D). The migrating A_2_B_5_+ cells with bipolar morphology were not in clusters and rather found scattered in the cortex of HP brains (Fig. 1B). Contrary to this, in the LP cortex, very few weakly stained A_2_B_5_+ cells were recorded (Fig. 1E). At E18 the A_2_B_5_+ GRP clusters and migrating population were clearly evident in the hilus region (Fig. 1C), while such cells were scant in the age-matched LP brains (Fig. 1F). This clearly indicates the compromised progenitor pooling as well as dissemination to other areas following maternal PMN.

Quantitation of relative immunofluorescence intensity of A_2_B_5_ (red) between LP and HP brain sections evidenced significantly low mean fluorescence intensity in LP at E14 [LP, 2.89±0.30 (mean±s.e.m.); HP, 12.17±1.99; *n*=8, *P*≤0.001], E18 (LP, 9.21±1.20; HP, 17.22±3.01; *n*=11, *P*≤0.023) and P0 (LP, 7.82.21±1.62; HP, 13.05±1.85; *n*=12, *P*<0.025) as shown in the histogram (Fig. 1G). With proceeding development, the A_2_B_5_ expression downregulation trend was recorded in HP controls with a decrease in expression from E18 onwards and a very few A_2_B_5_+ cells were seen after P5, evidencing that the transitional states of A_2_B_5_+ progenitors shunting towards the glial lineage are short lived.

### BLBP immunoreactivity revealed low progenitor population and loss of colonization and proliferative potential following maternal PMN

**Fig. 2.**
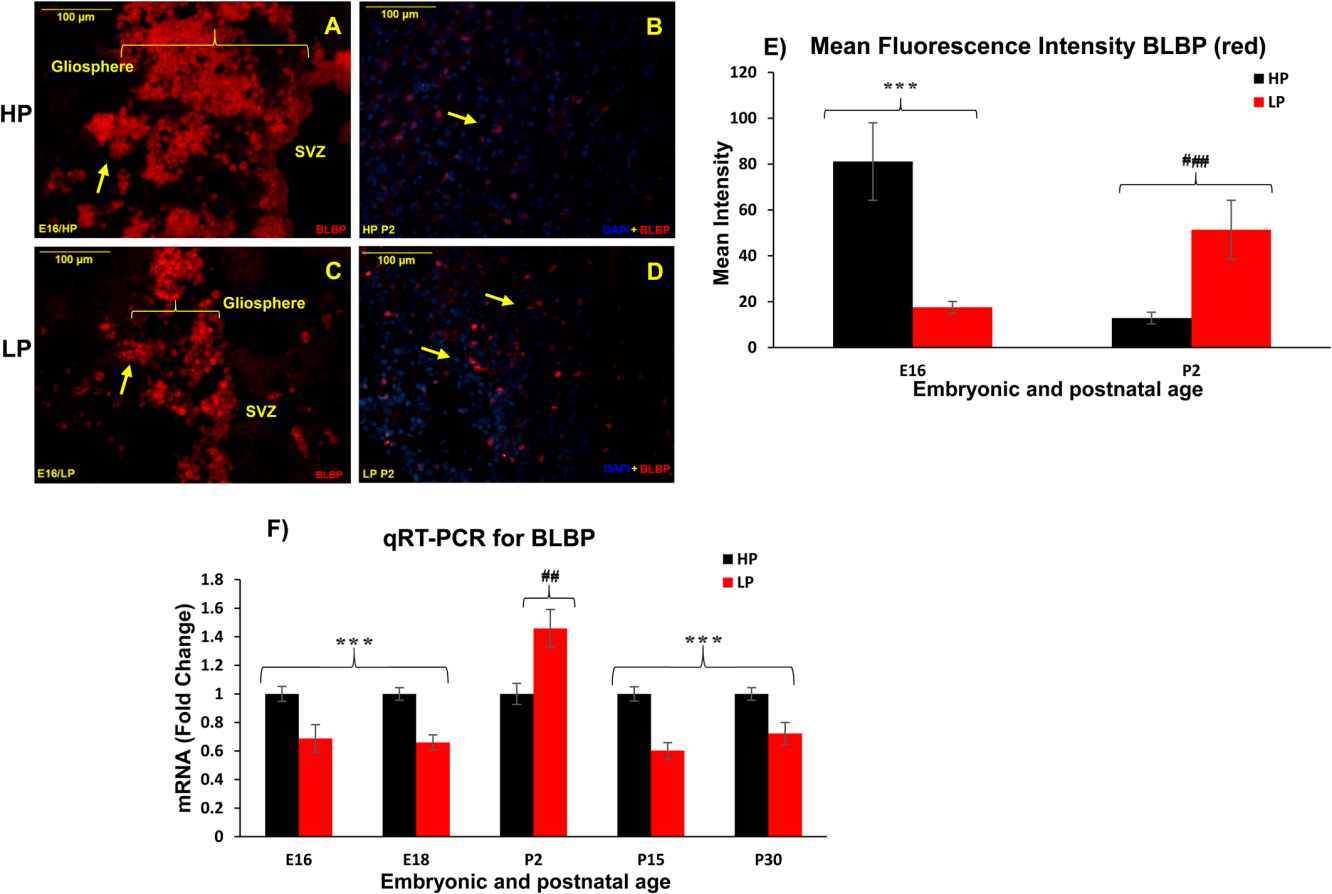
**Loss of colonization and low BLBP+ progenitor population in neurogenic and gliogenic niches of LP F1 embryonic brains.** Photomicrographs showing BLBP (red) immunolabelled secondary progenitor pool, contrary to abundant BLBP+ cells and large sized gliospheres in E16 HP brain (A), age-matched LP brains presented dissociated BLBP+ progenitors and low clustering/gliosphere formation (C). A significant increase in BLBP immunoreactivity was noticed in LP P2 brains (D) with respect to age-matched HP controls (B). Yellow arrows indicate BLBP+ clusters/cells; square brackets in A and C indicate gliospheres. Scale bars: 100 µm. (E) Mean fluorescence intensity of BLBP (red) represented as histogram shows significantly decreased BLBP expression in LP counterparts at E16 with surprising increase at P2 as compared to HP controls. Error bars indicate s.e.m.; ***P≤0.001; ^###^*P*≤0.001 (Student’s *t*-test). (F) Quantitative real-time RT-PCR results also evidenced significantly reduced BLBP fold expression at all study time points in LP brain samples except for P2 validating the immunohistochemical findings. Graph shows the fold expression±s.e.m. (*n*=3) in LP normalized to 1 in the HP. ***P≤0.001; ^##^*P*≤0.02 (Student’s *t*-test).

BLBP, a small nucleocytoplasmic protein, is considered as a marker for secondary radial glial cells that will give rise to astrocytes. The BLBP+ radial progenitors persisted throughout the neurogenic and gliogenic phases of temporal brain development with a peak increase from E15-18, marking the gliogenesis period. BLBP is primarily expressed in immature astrocytes that downregulate BLBP late in their differentiation phase. At E16, in HP group preparations, a robust BLBP expression was detected in the radial progenitors that were found clustered in the SVZ forming prominent well-spaced gliospheres (Fig. 2A). In addition, a small number of BLBP+ radial progenitors were also localized in the hippocampus, thalamic regions, midbrain and pre-optic areas. Contrary to this, a significant reduction in number of the BLBP+ progenitors and expression intensity was recorded in the LP group both in the ventricles and the neighbouring areas. More so, the BLBP+ progenitors either remained in the discrete form or clustered into small gliospheres (Fig. 2C), speculating loss of colonization potential. However, a surprisingly increased expression of BLBP was noticed in LP brains at P2 (Fig. 2D) as compared to age-matched HP brains (Fig. 2B).

Measurement of relative fluorescence intensity of BLBP with NIH Fiji Image J software confirmed the above results with a significantly decreased mean fluorescence intensity at E16 (LP, 17.54±2.57; HP, 81.10±16.88; *n*=8, *P*= 0.004) and an increased mean intensity at P2 in LP brains with respect to age-matched HP controls (LP, 51.38±12.85; HP, 12.87±2.5; *n*=8, *P*<0.001; Fig. 2E). The real time RT-PCR data further confirmed this, showing a significantly low BLBP mRNA fold expression at E16 (LP, 0.68±0.09; *n*=4, *P*< 0.02) and E18 (LP, 0.65±0.05; *n*=3, *P*≤0.002) and subsequent upregulation at P2 (LP, 1.42±0.13; *n*=4, *P*<0.02) in LP brains normalized to age-matched HP controls (Fig. 2F), suggesting a compensatory response. However, with subsequent development the BLBP mRNA levels remained significantly low both at P15 (LP, 0.60±0.05; *n*=3, *P*<0.002) and P30 (LP, 0.72±0.07; *n*=3, *P*<0.02) in LP F1 brains indicating an overall decrease in quantitative fold expression of BLBP mRNA following PMN (Fig. 2F).

### Delayed emergence of GFAP, early maturation and low astrocytic density evidenced following intra-generational protein malnutrition

**Fig. 3.**
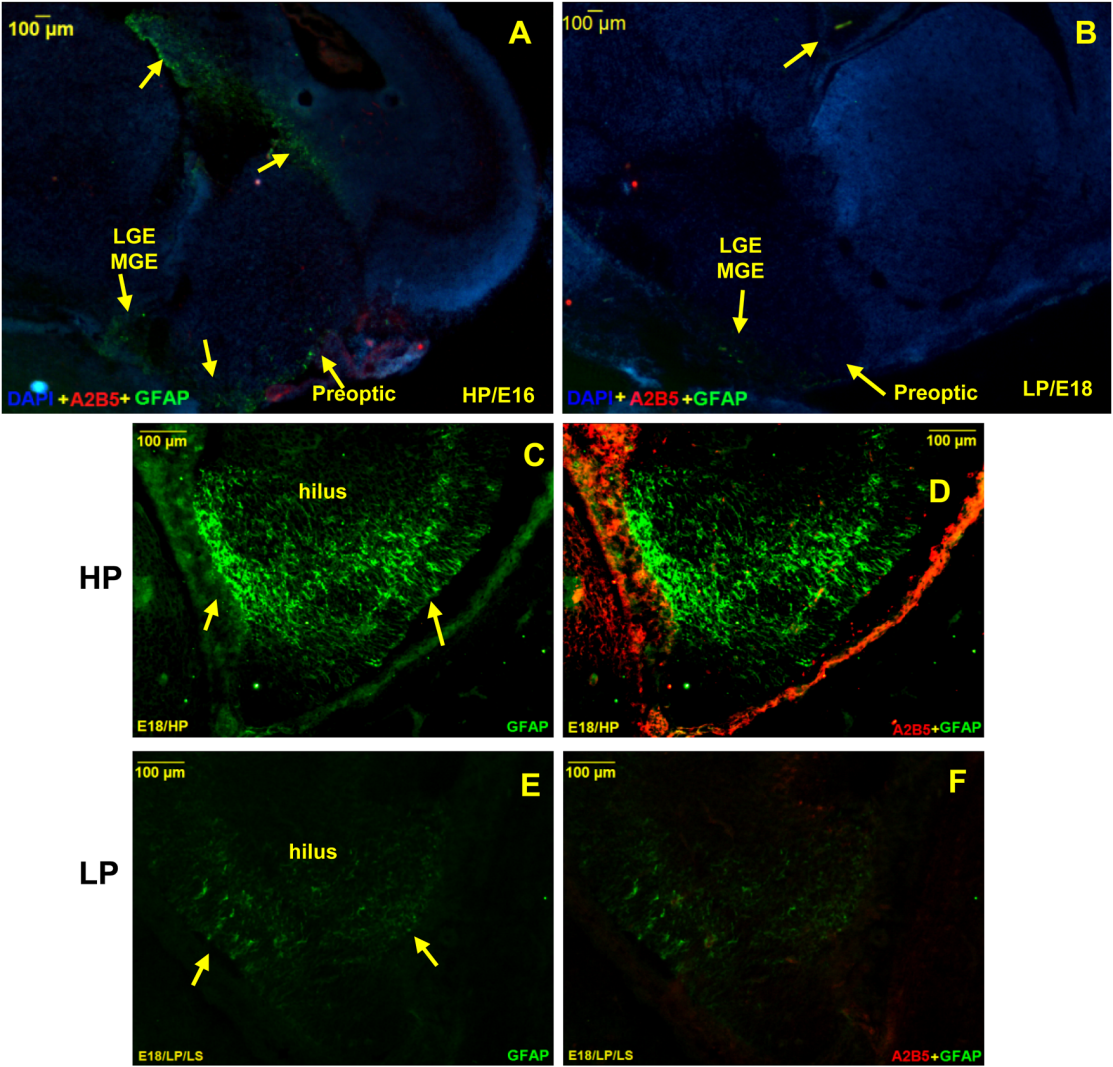
**Delayed emergence of GFAP in LP embryonic brain.** Photomicrographs showing dual immunofluorescence labelling of anti- A_2_B_5_ (red) and anti-GFAP (green) in brains at E16 (A) and E18 (B). GFAP emergence is clearly evident (yellow arrows) in HP E16 brains along the pre optic areas and lateral and medial ganglionic eminence, however, a mild expression of GFAP was noticed in LP brains only at E18 (yellow arrows) suggesting delayed astrogenesis in LP brains. Higher magnification images from HP E18 brains through hilus clearly show abundant GFAP+ astrocytes (yellow arrows; C) with significantly low astrocyte number in LP counterparts (yellow arrows; E). Merged image from HP E18 brain (D) shows a robust A_2_B_5_ expression with abundant GFAP+ astrocyte population with negligible A_2_B_5+_ GRP number and scanty GFAP+ astrocyte population (yellow arrows) in age and area matched LP brains (F). Note that HP images in C and D are the same section as that shown in Fig. 1C. Scale bars: 100 µm.

**Fig. 4.**
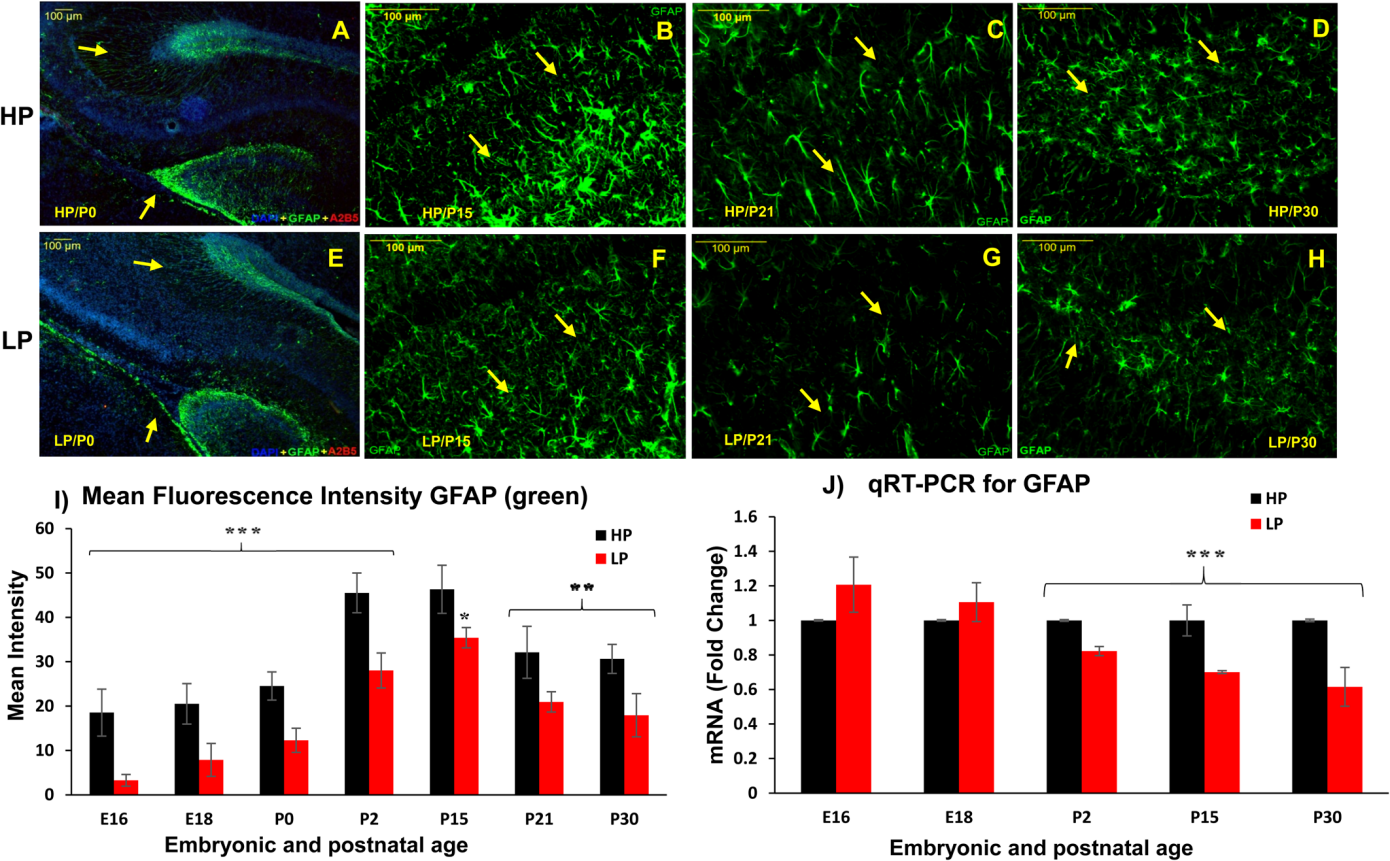
**Diffused and disorganized glial tubes with significantly low astrocyte population speculates compromised astrogenesis following PMN.** Photomicrographs of brain sections at postnatal day 0 (A,E), 15 (B,F), 21 (C,G) and 30 (D,H) from LP and HP group animals immunolabelled with anti-GFAP (green). Diffused and disorganized glial tubes are clearly evident (E, yellow arrows) with low GFAP expression in LP brains at birth as compared to age-matched HP controls (A). Representative images showing a significantly low GFAP+ astrocyte population in LP brains at P15 (F), P21 (G) and P30 (H) with respect to age-matched HP controls (B, C and D, respectively). Yellow arrows indicate GFAP+ astrocytes. Scale bars: 100 µm. (I) Mean fluorescence intensity measurements of GFAP (green) show relative changes in immunoreactivity at different study time points supporting significantly decreased GFAP expression in protein-malnourished rat brains from E16- P30. Graph shows mean±s.e.m. (J) Graph showing quantitative fold change in temporal GFAP mRNA expression in LP and HP brain samples through real time RT-PCR, evidencing significantly decreased GFAP expression in LP brains from P2-P30 and non-significant increase at embryonic days 16 and 18. Graph shows the fold expression±s.e.m. (*n*=3) in LP normalized to 1 in the HP. **P≤0.03, ***P≤0.001 (Student's *t*-test).

Immunofluorescence studies revealed appearance of GFAP first in the hippocampal and pre-optic regions in HP brains at E16 (Fig. 3A), followed by other areas. Such emergence of GFAP was delayed in the LP embryos and could only be seen at E18 (Fig. 3B) as opposed to E16 in the HP group (Fig. 3A). GFAP expressing differentiated astrocytes with typical astrocyte morphology were clearly seen by E18 in the hilus region of HP brains (Fig. 3C), however, a significantly low GFAP immunoreactivity was noticed in LP E18 brains (Fig. 3E). The dual immunolabelling of A_2_B_5_ and GFAP clearly indicate a direct relation between the population of A_2_B_5_+ GRPs and GFAP+ astrocytes. This is clearly evident from our images showing dense population of A_2_B_5_+ GRPs and GFAP+ astrocytes in the hilus region of HP E18 brains (Fig. 3D) and negligible A2B5+ GRPs and scant astrocyte population in the age-matched LP brains (Fig. 3F). Although, GFAP immunoreactivity was localized mainly to the marginal cells of hippocampus in HP brains by E18, from P0 onwards, radial and typical ramified GFAP+ cells were seen in all hippocampal subfields and also other brain regions (Fig. 4A). As in the brains of newly born rat (P0), the GFAP+ progenitors and immature astrocytes continue to proliferate and migrate radially towards the cortex forming astroglial tubes, such glial tubes, and were diffused and disorganized in LP brains (Fig. 4E) as compared to the well-organized glial tubes in HP counterparts (Fig. 4A). An abrupt increase in GFAP+ astrocyte number to several folds was noticed by P2 and peaked by P15-30 in HP brains (Fig. 4B-D). However, a significantly low GFAP+ astrocyte density was recorded in age matched LP brains (Fig. 4F-H).

**Fig. 5.**
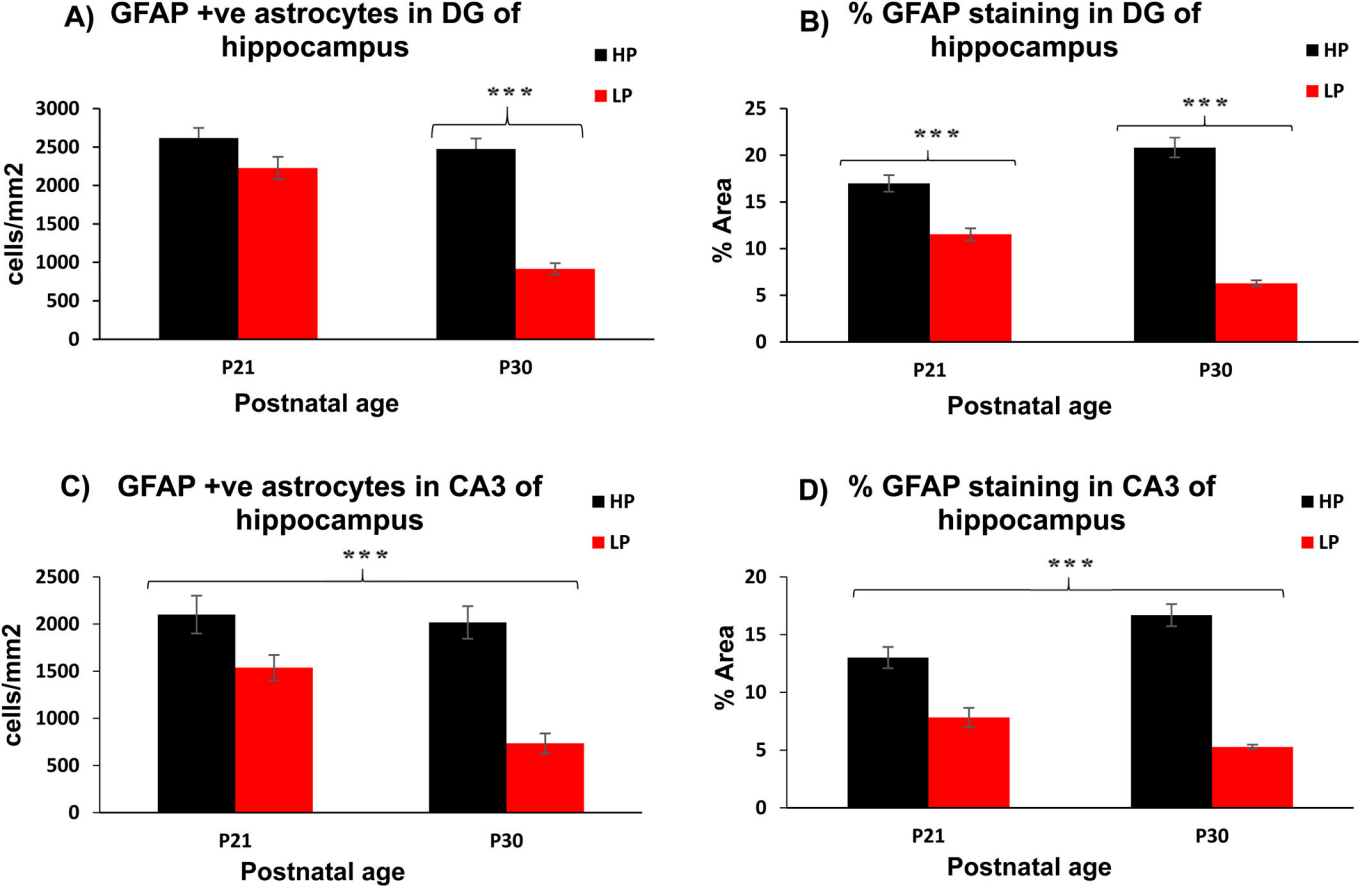
**Compromised astrocyte density and GFAP expression following maternal PMN.** Leica interactive cell quantification data of GFAP+ astrocytes and % GFAP immunostaining in DG and CA3 subfields of P21 and P30 rat hippocampus reveals a significantly low astrocyte count (A,C) and significantly reduced % GFAP (B,D) staining in LP brain sections with respect to age matched HP brains. Values are expressed as mean±s.e.m. (cell count/frame, A and C) and % GFAP staining (B and D). ***P≤0.001 (Student's *t*-test) for comparison of LP F1 group with respect to HP F1 controls.

The GFAP immunofluorescence (green) quantification represented in histogram (Fig. 4I) revealed significantly low mean fluorescence intensity in LP brain preparations at E16 (LP, 3.27±1.32; HP, 18.54±5.29; *n*=10, *P*< 0.01), E18 (LP, 7.87±3.60; HP, 20.50±4.41; *n*=12, *P*<0.03), P0 (LP, 12.29±2.73; HP, 24.53±3.12; *n*=9, *P*<0.001), P2 (LP, 28.03±3.94; HP, 45.51±4.47; *n*=8, *P*<0.01) and P30 (LP, 17.94±4.86; HP, 30.64±3.27; *n*=8, *P*<0.025). Results from qRT-PCR assay also evidenced consistently low GFAP mRNA levels in the hippocampus of LP brain samples, except for an insignificant increase at E16 and E18. However, at P2 (0.8±0.02; *n*=3, *P*≤0.001), P15 (0.70±0.08, *n*=3, *P*≤0.001) and P30 (0.61±0.11, *n*=3, *P*≤0.001), the GFAP expression was significantly low in the LP brains with expression normalized to age-matched HP brains (Fig. 4J). Leica QWin interactive cell quantitation data of GFAP+ astrocytes also revealed that maternal PMN leads to a highly significant decrease in astrocyte density at P30 in dentate gyrus (DG) (LP, 915.25±74.24; HP, 2475±136.50; *n*=12, *P*<0.001, Fig. 5A) and at P21 and P30 in CA3 (P21; LP, 1537.50±135.21; HP, 2100.50±200.12; *n*=12, *P*<0.001, P30; LP, 735.02±104.46; HP, 2017.50±172.50; *n*=12, *P*<0.001, Fig. 5C) subfields. In addition, the percent immunostaining of GFAP was also significantly decreased at P21 and P30 in both DG (P21; LP, 11.53±0.64; HP, 16.98±0.88; *n*=12, *P*<0.001, P30; LP, 6.28±0.31; HP, 20.81±1.04; *n*=12, *P*<0.001, Fig. 5B) and CA3 (P21; LP, 7.82±0.83; HP, 13.0±0.91; *n*=12, *P*<0.001, P30; LP, 5.27±0.25; HP, 16.68±0.95; *n*=12, *P*<0.001) regions of LP brains (Fig. 5D). This further confirms the above findings evidencing compromised astrogenesis following maternal PMN.

### Precocious differentiation and maturation of astrocytes following maternal protein malnutrition

**Fig. 6.**
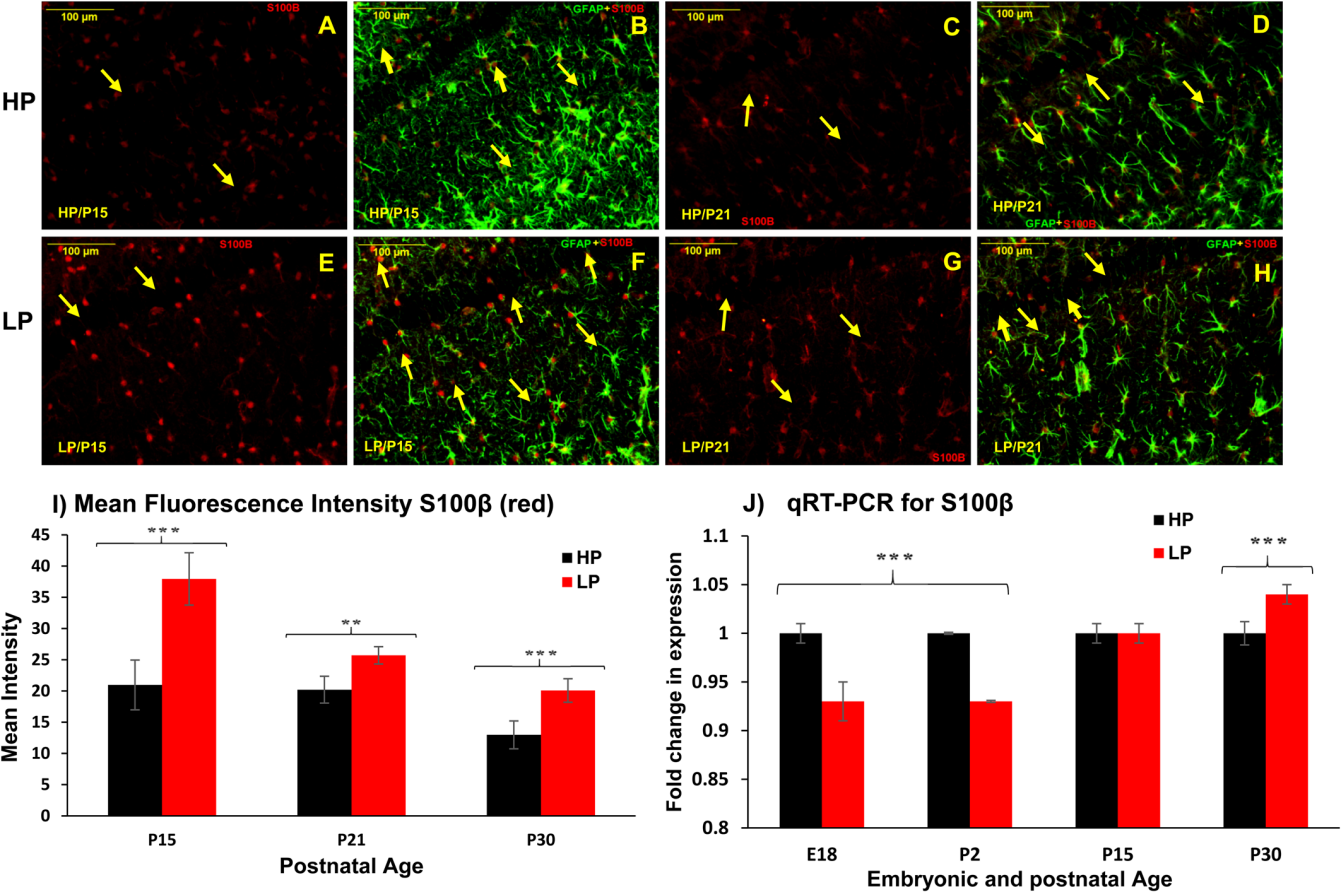
**Early S100β expression in GFAP+ astrocytes speculates precocious astrocyte maturation.** Dual immunolabelling with S100β (red) and GFAP (green) reveal a significant increase in S100β+ cell number in LP brain preparations at P15 (E) and P21 (G) as compared to age matched HP controls (A and C). Merged images reveal that almost all GFAP+ astrocytes co-express S100β+ as well in LP brains (F,H), contrary to very few GFAP+S100β co-expressing astrocytes in HP brain (B,D) supporting precocious astrocyte maturation in LP brains. Note that HP images in B and F are of the same section as shown in Fig. 4B,F. Scale bars: 100 µm. (I) Mean fluorescence intensity measurements of S100β (red) through Fiji ImageJ also evidenced significantly increased S100β expression in LP counterparts. (J) The fold change in expression of S100β through real time qRT-PCR revealed significant downregulation at E18 and P2 in LP brain hippocampi with increased expression at P30, when normalized to HP controls. Error bars in I and J indicate s.e.m. **P≤0.025, ***P≤0.001 (Student’s *t*-test).

The expression of S100β in brain is developmentally regulated. Although the radial glial cells in the SVZ express S100β during early development, this protein is downregulated during late embryonic development (Patro et al., 2015). The immature astrocytes in the early postnatal brain remain S100β negative and the protein is re-expressed during the first postnatal week, when astrocytes start to mature. S100β, thus act as a marker of differentiation and maturation of astrocytes during normal development. Immunohistochemical localization of S100β revealed high S100β+ cells in LP brains at P15 (Fig. 6E) and P21 (Fig. 6G) as compared to HP counterparts (Fig. 6A and C, respectively). From merged images it was clearly evident that almost all GFAP+ astrocytes co-expressed S100β at P15 in LP brain sections (Fig. 6F, yellow arrows) as compared to very few in age-matched HP (Fig. 5B) indicating precocious astrocyte maturation following maternal PMN. At P21 as well, more GFAP+/S100β+ mature astrocytes were seen in LP brains (Fig. 6H) with respect to HP controls (Fig. 6D).

Mean fluorescence intensity measurements using Fiji ImageJ also evidenced significantly increased S100β expression at P15 (LP, 37.95±4.20; HP, 20.97±3.98; *n*=12, *P*<0.005), P21 (LP, 25.71±1.38; HP, 20.97±3.98; *n*=8, *P*<0.025) and P30 (LP, 20.08±1.89; HP, 12.97±2.23; *n*=11, *P*<0.02) in LP brains as compared to age-matched HP counterparts (Fig. 6I) validating the immunohistochemical findings. Data from the qRT-PCR assay evidence a significant decrease in S100β fold expression at E18 (LP, 0.93±0.02; *n*=4, *P*=0.035) and P2 (0.92±0.01; *n*=3, *P*<0.025) in LP brain samples with expression normalized to age-matched HP controls. Subsequently, a significant fold increase in S100β expression was recorded in LP brains at P30 (LP, 1.04±0.01; *n*=3, *P*<0.04; Fig. 6J) suggesting astrogliopathy, as S100β overexpression during late postnatal development is an established marker of gliopathy.

The inconsistency of the qRT-PCR data and the antibody results for some gliogenesis markers could rather be because of the region-specific protein expression. For qRT-PCR, RNA isolation was done from hippocampal tissue only, while the immunohistochemistry was performed in the whole brain sections. The future endeavour of the authors would be to study this bias by region-specific expression analysis of gliogenesis markers.

## DISCUSSION

The neuron-centric doctrine has dominated neuropathology for a long time, and neuronal death, damage and other pathophysiological events were generally emphasized as causatives. However, recent studies have established an imperative role of glia in neurodevelopmental and neuropsychiatric illnesses (reviewed by Verkhratsky and Parpura, 2016). During embryonic and early postnatal development, astrocytes play a crucial role in neuronal migration, axonal outgrowth, formation, maturation and remodelling of synapses and information processing (Shu and Richards, 2001; Pfrieger, 2002; Verkhratsky et al., 2013). The more advanced roles of astrocytes in behaviour (Oliveira et al., 2015), neural circuit development (Clarke and Barres, 2013) and other higher order functions have indicated astrocytes as candidates in neuropsychiatric illnesses. The developing brain is highly susceptible to environmental insults like protein deprivation, infections and other stresses during *in utero* and early postnatal periods and has severe and permanent consequences. PMN induced adverse effects in the developing brain have largely focused on neurons, and only a few studies have investigated the glial changes (Clos et al., 1982; Feoli et al., 2008; Chertoff, 2015). The role of astrocytes in various brain disorders is being increasingly established with a limited knowledge of astrogenesis following developmental challenges. To the best of our knowledge, no complete study elucidates temporal and spatial astrogenesis following stressor effect, and this incited us to assess the impact of intra-generational protein malnutrition on the astrogenesis in the developing rat brain.

Astrogenesis commences within the brain during late embryogenesis shortly after the peak neurogenesis period is over. The generation of abundant astroglia mainly involves three sources: GRPs that get distinguished from NEP's during E11-12 by the expression of A_2_B_5_; secondary radial glia and astrocyte precursors of SVZ marked by BLBP expression; and the local proliferation of differentiated astrocytes in the postnatal cortex (Rowitch et al., 2002; Bayraktar et al., 2015). A drastically low A_2_B_5_+ GRP population observed in the ventricles and cortices of LP brains in present study clearly evidence a compromised progenitor pooling with a reduction in the dissemination of these GRPs to cortex and sub-cortical structures following maternal PMN.

Another major source for astrogenesis is the BLBP+ secondary RG's of SVZ. This study indicates abundant BLBP+ progenitors residing in the ventricles during early embryogenesis and later in SVZ of the HP brains with peak expression at E14-16. Similar to A_2_B_5_+ GRPs, LP brains presented low BLBP immunoreactivity, both in terms of expression and progenitor number. A low protein diet leads to consistent loss of colonization of BLBP+ clusters suggesting low proliferative potential during early embryogenesis, i.e. E14-16. Contrary to the large-sized gliospheres and BLBP+ cell clusters of HP brains, LP brains presented discrete BLBP+ cells with small or no clustering. As the BLBP+ progenitors are responsible for the production of abundant glia, astrocytes, in particular during the late embryonic period, reduce in progenitor number and their proliferative potential clearly reflects the compromised astrogenesis in LP rats. The significant increase in BLBP expression at P2 in the LP brain points to a delayed but compensatory phenomenon against reduced astrogenesis in LP brains.

Expression of GFAP, an astrocyte signature protein, acts as a marker of terminally differentiated astrocytes (Allaman et al., 2011). In accordance with the earlier publications (Miller et al., 1985; Liu et al., 2002), the present study reports that GFAP-expressing cells were observed by E16 in areas like pre optic, lateral and medial ganglionic eminence, pallium and sub pallial structures in HP brain, while in the LP brain no GFAP expression was noted until E18, indicating a significant delay. Abundant and discrete star shaped GFAP+ astrocytes were noticed in HP E18 brains especially along the hippocampal formation and hilum areas indicating their gradual maturation and elaboration of processes, which was completely absent in the LP brain supporting a compromised and/or delayed astrogenesis and reduced population. A significantly high A_2_B_5_ and GFAP expression in hilum region of HP brain further supports the abundant astrocyte formation as this represents another proliferative zone around birth. BLBP expression in the SVZ progenitors stimulate their migration and proliferation and BLBP downregulation is necessary for these cells to differentiate as astrocytes. In rat brain, by early postnatal stages the proliferation and diversification of astrocytes is largely complete, however, the elaboration and refinement of astrocytic processes continues well into the postnatal period, coinciding with the period of active synaptogenesis pertaining to their role in promoting synapse formation. Such sequential developmental changes in the astrocytes leads to the maturation of astrocytes marked by a gradual upregulation in the expression of GFAP, Aquaporin-4 and S100β (Molofsky et al., 2012). Reduction in postnatal glial cell density has also been reported in some mouse models of restrain stress in hippocampus or other regions of the brain associated with stress-related behaviour (Leventopoulos et al., 2007; Behan et al., 2011).

Early expression of S100β in the GFAP+ astrocytes observed in the present study, with almost all GFAP+ astrocytes co-labelling S100β at P15 in LP brains, indicates precocious astrocyte maturation. However, such coexistence of GFAP/S100β was observed in HP brain only at P30. S100β, a calcium binding protein, has been implicated in the regulation of microtubule assembly of type III intermediate filaments and is involved in the cell proliferation and differentiation (Raponi et al., 2007; Donato et al., 2013). In astrocyte development, the S100β expression defines a late developmental stage after which GFAP expressing astrocyte precursors lose their stem cell potential and acquire a more mature developmental stage (Raponi et al., 2007). This provides a clue for the interpretation of the present results stating the precocious differentiation and early morphological maturation of astrocytes following PMN. This further explains that the low astrocyte number and density at P30 reported in this study in LP brains could be resultant of such precocious differentiation, as the precursors lose their proliferative potential and exit the cell cycle much earlier than the usual spatial temporal timeline. Keeping in view the well-established correlation of high S100β levels in many neurodegenerative diseases like Parkinson's (Liu et al., 2008, 2011), Alzheimer's (Wilcock and Griffin, 2013), hypoxia ischemia (Wainright et al., 2004), experimental autoimmune encephalitis (Yan et al., 2003), mood disorders (Schroeder et al., 2010) and Schizophrenia (Rothermundt et al., 2009), an early and enhanced expression of S100β in the astrocytes of protein malnourished rat brain could also be one of the factors leading to the behavioural and cognitive impairments reported in our earlier publication (Naik et al., 2015).

Taken together our results evidence a reduced number of A_2_B_5_+ GRPs both in ventricles and migrating to the cortex, low BLBP+ progenitor pool and their loss of colonization and significantly low differentiated astrocyte population in LP brain. This further strengthens the hypothesis of compromised astrocyte development in the LP model. Notwithstanding the functional and imperative role of astroglia in physiology and pathophysiology, compromised astrogenesis in the LP model speaks volumes about the impaired development and is rather a reason for the abnormal functioning at levels of behavioural and cognitive aspects. This is in line with our earlier report indicting hyperactivity, poor learning and memory, and impaired habituation in the PMN model (Naik et al., 2015). Recently reduced expressions of GFAP and low density of GFAP+ cells has been reported in major depression, linking neuropsychiatric illnesses and astrocytes (Cobb et al., 2016). A central role of astrocyte degeneration induced homeostatic failure as fundamental for the initiation and progression of neuropathological diseases like infections, amyotrophic lateral sclerosis, schizophrenia, and autism have also been evidenced (Laurence and Fatemi, 2005; Kolomeets and Uranova, 2010; Schnieder and Dwork, 2011; Valori et al., 2014; Sharma et al., 2016). Other reports show a reduction in GFAP levels and decreased glial density in the prefrontal cortex, cortico-limbic areas and amygdala in both the adult brain of patients with psychiatric disorders and mouse models (Gosselin et al., 2009; Altshuler et al., 2010).

Keeping in the view the role of astrocytes in synaptogenesis, synapse stabilization and elimination as well as myelination via aligning oligodendrocyte precursors with axons, it is undoubtedly clear that these are the crucial elements for neural circuit formation during brain development. More recent gliocentric approaches to neuropsychiatric diseases and analysis of neurodevelopmental disorders all point towards the astrocyte dysfunction during development resulting in disease progression and pathology (Molofsky et al., 2012; Sloan and Barres, 2014). Although we have not come across any study reporting a developmental delay in astrocytogenesis as recorded in our study following PMN, alterations of timing in terms of precocious astrogenesis has been implicated in the pathology of many genetic diseases involving neurocognitive delay, like Rasopathies (Gauthier and Bukach, 2007; Tidyman and Rauen, 2009; Paquin et al., 2009) and Down's syndrome (Zdaniuk et al., 2011; Lu et al., 2011). As the brain development is a temporally extended and complex process requiring the synthesis of cellular components such as nucleic acids and proteins for various developmental events associated with neurogenesis and gliogenesis, the delay in the LP brain could be due to the prolonged neurogenic phase as a compensatory neurogenesis, which could be further studied. The sustained protein deprivation throughout the embryonic and postnatal life leads to the long lasting and permanent deficits in the offspring. Delta notch and a variety of transcription factors have been implicated to have a role directly or indirectly in astrocytogenesis, regulation of precursor pool size, proliferation and gliogenesis (Gaiano and Fishell, 2002; Pierfelice et al., 2011). Both the temporal delay in the appearance and total number of GFAP-positive astrocytes following PMN suggest that maternal protein deprivation induces both the change in size of the progenitor pool as well as the timing of neuron to glia switch. As both time and space appear to be the chief regulators of primary progenitors in the brain, it would be important to further expand our understanding for the mechanism of astrocyte dysfunction in the PMN brains and also to design future therapeutic strategies. In our earlier publication using the same model (Naik et al., 2015), we reported the behavioural and cognitive impairments in the F1 offspring which are evident and consistent with the cellular and molecular changes reported in this investigation. Thus, it is proposed that the offspring born to protein-malnourished mothers are at a higher risk of developing neuropsychiatric disorders at adolescence or at a later age may be due to developmental astrocytic abnormalities.

### Conclusions

**Fig. 7.**
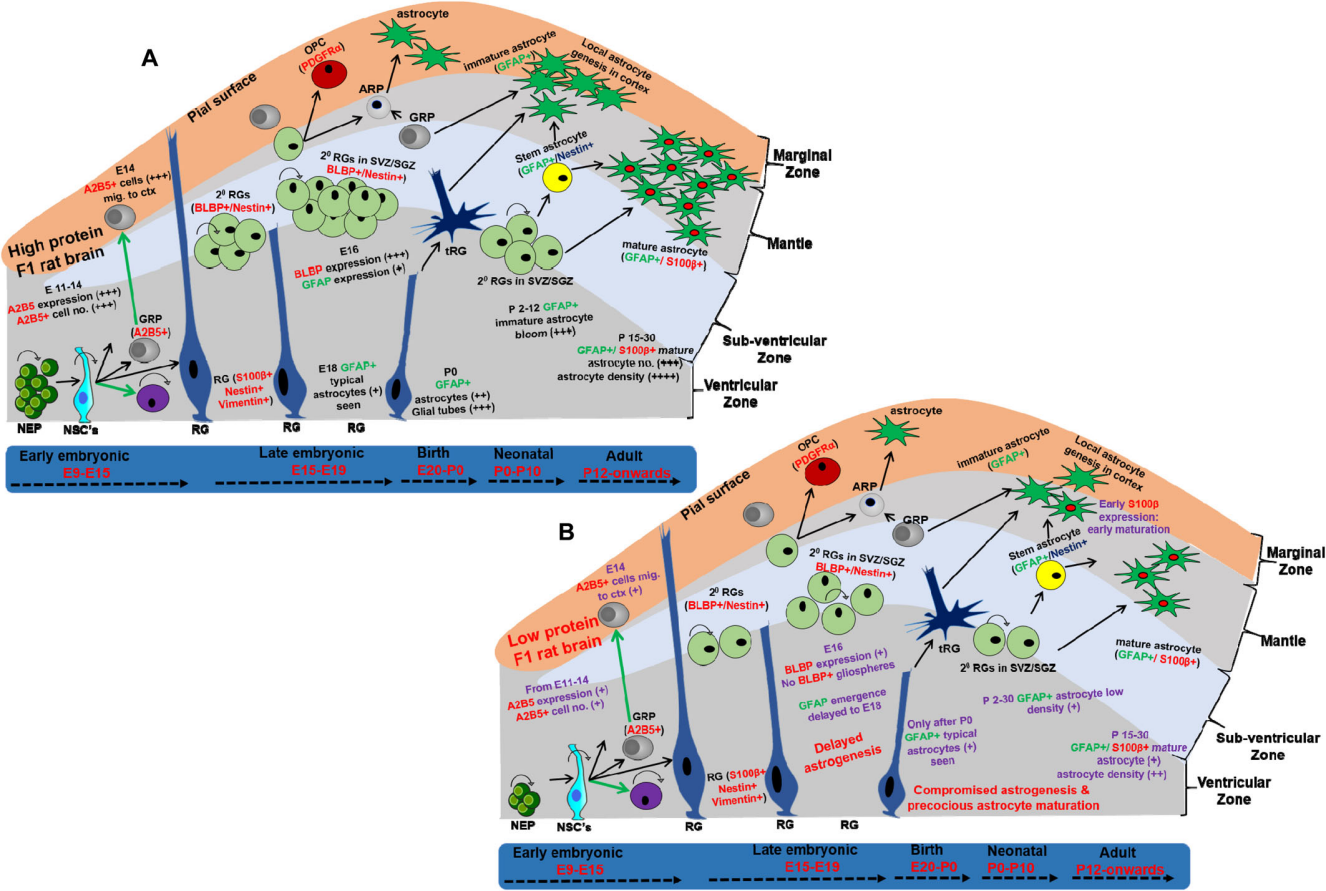
**Picturesque representation of progenitor heterogeneity and temporal astrogenesis in HP and LP brains.** (A) HP brain, (B) LP brain. PMN induced impairments in temporal astrogenesis are shown in violet text in B. +, low; ++, medium; +++, high; ++++, very high; NEP, neuroepithelial cell; NSC, neural stem cell; SVZ, sub-ventricular zone; SGZ, sub-granular zone; GRP, glial restricted precursor; ARP, astrocyte restricted precursor; RG, radial glia; tRG, transforming RG; OPC, oligodendrocyte precursor; 2° RGs, secondary RGs; E, embryonic day; P, postnatal day; LGE, lateral ganglionic eminence; MGE, medial ganglionic eminence.

The spectrum of data gathered indicate that intra-generational protein malnutrition alters foetal programming, with respect to low progenitor pool, impaired temporal astrogenesis, low astrocyte density and precocious astrocytic maturation in the LP-F1 progeny (Fig. 7). The findings of the present study clearly reflect the immediate relevance to the hypothesis of astrocytes as possible drivers of neurodevelopmental dysfunction. The outcome of this study will increase our understanding of the early life stressor associated brain development/glial dysfunction and consequent pathophysiology, and would provide new targets and windows for effective therapeutic intervention against debilitating neurological disorders.

## MATERIALS AND METHODS

### Animal model

Nulliparous Sprague Dawley (SD) female rats (160-180 g, 2 months old) were housed under standard laboratory conditions in a 12 h:12 h light:dark cycle at 23±2°C room temperature with *ad libitum* access to either of the two diets: (i) low protein (LP, 8% protein, *n*=8), or (ii) high protein (HP, 20% protein, *n*=8) obtained from National Institute of Nutrition, Hyderabad, India (Table 1). The naïve SD females were switched to either of the two diets 45 days before pregnancy and continued on same diets until the last study time point. Timed pregnancies were set in the dams by a 4 h pairing with males. The stage of oestrous with sperm was assessed by light microscopic examination of cells obtained from vaginal smears collected before 9:00 h every morning and if positive, females were designated as gestational day 0 (GD0). Timed pregnant females were observed carefully every 2 h on the expected days of delivery to mark the day of birth as P0. Post weaning, the LP and HP pups were housed three per cage and maintained on the same diet until the termination of the experiment.

**Table 1.**
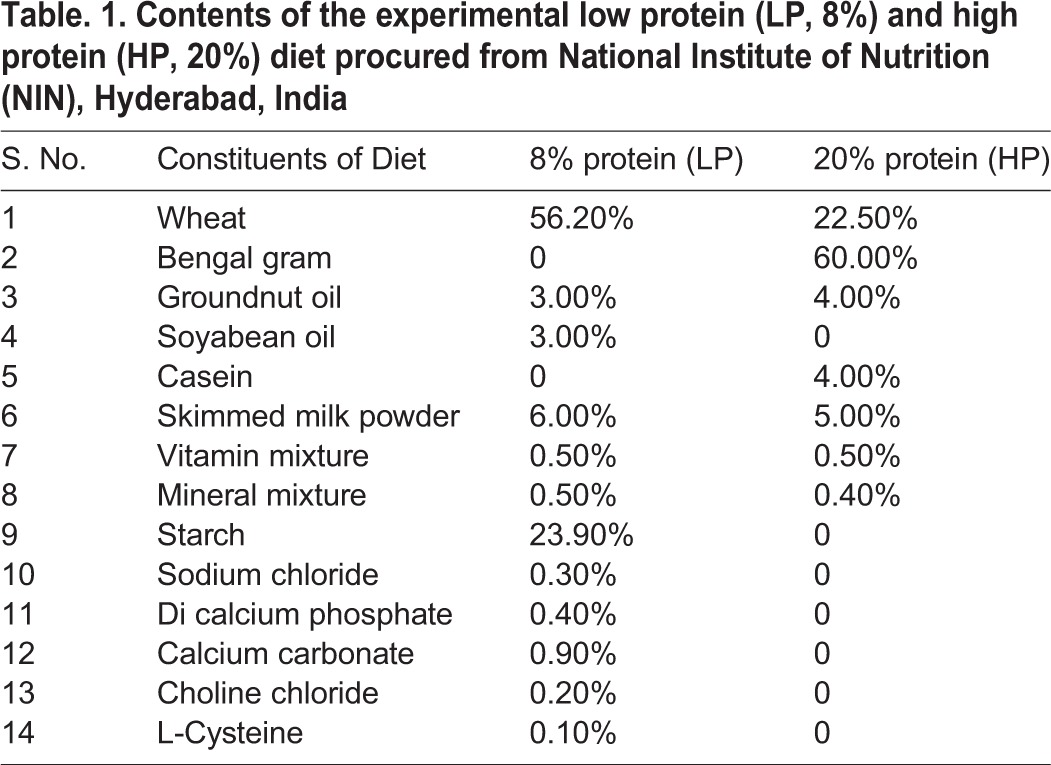
**Contents of the experimental low protein (LP, 8%) and high protein (HP, 20%) diet procured from National Institute of Nutrition (NIN), Hyderabad, India**

### Embryo and foetus harvesting

The pregnant females from the respective LP and HP groups were deeply anesthetized with diethyl ether and the embryos/foetuses of varied embryonic ages (E11, 14, 16 and 18) were excised surgically with atraumatic measures. The brain tissues of the embryos were micro dissected. Half of the tissues were stored in RNA Later solution (Sigma, USA) for RNA isolation, while other half was processed for cryosectioning. Embryonic tissues for cryosectioning were fixed in 2% paraformaldehyde (PFA) in 0.01 M PBS, pH 7.4 for 24 h, followed by 3 times washing in phosphate buffer and subsequently cryoprotected with sucrose gradients (10%, 20% and 30%).

### Postnatal brain harvesting

The pups born to LP and HP females were sacrificed at the respective timepoints (P0, P2, P6, P12, P15, P21 and P30) for harvesting brain tissue. The animals were deeply anesthetized and perfused transcardially with ice-cold saline followed by 2% PFA in 0.01 M PBS, pH 7.4. The brains were dissected out and post-fixed overnight with 2% PFA. The tissues were subsequently cryoprotected with sucrose gradients (10%, 20% and 30%) prepared in 0.01 M PBS. Sections of 14 µm thickness were cut with the help of Lieca Cryotome (CM1900; Germany) and collected on chromalum gelatin-coated slides. For embryonic brains the sections were cut sagittally, while for postnatal brains the coronal sections were cut through the occipito-temporal region. The sections were stored at −20°C to be used for immunohistochemical studies. All the experiments were performed with prior approval and in accordance with the Institutional Animal Ethics Committee of Jiwaji University, Gwalior (M.P), India.

### Quantitative real-time assays

To assess the impact of intra-generational protein malnutrition on the dynamic changes in mRNA expression, levels of various marker genes of gliogenesis cycle, embryonic and postnatal brain samples of E16, E18, P2, P15 and P30 stored in RNA Later (Sigma, USA) were washed in autoclaved phosphate buffer saline and the hippocampus was micro dissected. Total hippocampal RNA was subjected to extraction using TRIzol^®^ (Life Technologies, 15596-018, USA) and total RNA was quantified using NanoDrop 1000 spectrophotometer (Thermo Scientific, USA) and run on gel to check integrity. A 500 ng of purified RNA was used for cDNA synthesis using the PrimeScript™ RT reagent kit (TaKaRa, RR047A, Japan). A 200 µg of resulting cDNA from all samples was used as template for PCR amplification in Applied Biosystems VIIA™7 Real-Time PCR system (Thermo Fischer Scientific, USA) using fast 96-well plates (Invitrogen, 4483354). Conditions used for real time PCR were as follows: 95°C for 10 min (1 cycle), 94°C for 20 s, 60°C for 20 s and 72°C for 30 s (40 cycles). Melt curves were generated to check the specificity of the annealing of primers to specific template. Results were analysed using comparative Ct method (2-[Δ][Δ]Ct). The gene-specific oligonucleotide primers for all the target genes as shown in Table 2 were designed using Primer Quest SciTool from Integrated DNA Technologies (IDT). All reactions were performed as *n*=3/sample (*n*=4 at E16 due to small volume of the brain) and in triplicates. Mean±s.e. was taken and presented as quantitative fold-change in expression. The differences in gene expression of various markers like BLBP, S100β and GFAP were performed using SYBR^®^ Green (Thermo Fischer Scientific) fluorogenic intercalating dye using 18S RNA as an internal control.

**Table 2.**
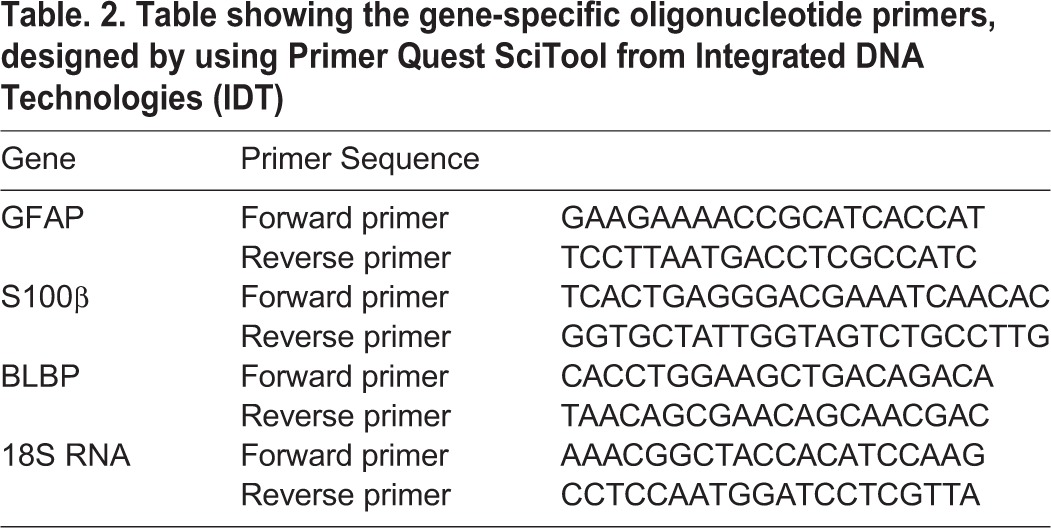
**Table showing the gene-specific oligonucleotide primers, designed by using Primer Quest SciTool from Integrated DNA Technologies (IDT)**

### Immunohistochemical labelling

A temporal and spatial analysis of development of astrocytes and their progenitors on foetal and pup brain slices from timed pregnant SD dams fed on experimental LP (8% protein) and HP (20% protein) diets was performed. The sagittal sections of foetal brain tissues and coronal brain slices from the postnatal brains were prepared for immunological staining. A change in GRP to the differentiated astrocytic lineage is accompanied by the expression of specific markers. To achieve this objective, a battery of immunohistochemical cell-specific markers was employed, A_2_B_5_ to distinguish GRPs from neuro epithelial cells (NEP's), BLBP as a marker of glia transforming, GFAP as astrocytic marker, S100β as marker of mature astrocytes and A_2_B_5_+BLBP, A_2_B_5_+GFAP, GFAP+S100β co-labelling to investigate any co-existence within the positive cells and lineage relationships. Negative controls were performed for each marker antibody by omitting the primary antibody. No specific labelling was seen in these sections. All the tissues were processed and stained in parallel to maintain comparability.

#### Immunostaining for GRPs and secondary radial glia

The cryocut brain sections from various stages of development *in utero* and postnatal life (E11, E14, E16, E18, P0, P2, P6, P15, P21 and P30) were air dried and then washed in PBS. The membrane permeabilisation was achieved by treating sections with 1% Triton X-100 in PBS for 20 min. The sections were subsequently washed thrice with PBST (0.5% Tween-20 added to PBS) and then incubated for 2 h with 10% normal goat serum (NGS) in PBS at room temperature for non-specific protein blocking. After blocking the sections were incubated overnight at 4°C with primary antibodies, i.e. anti- A_2_B_5_ (1:200, Mouse monoclonal, Abcam ab53521) or anti-BLBP (1:300, Rabbit polyclonal, Abcam ab32423). The binding of the primary antibodies, i.e. anti-A_2_B_5_ and anti-BLBP was visualized using goat raised TRITC labelled anti mouse (1:200, Sigma) and anti-Rabbit (1:200, Sigma) antibodies respectively. Both the primary and secondary antibodies were diluted in 5% BSA in PBS with 0.5% Tween-20. Control for immune labelling was performed with the same procedure without the primary antibodies. The sections after thorough washing with PBS were finally cover-slipped with Vectashield Hard+Set mounting medium with DAPI and visualized under the fluorescence microscope.

#### A_2_B_5_+ GFAP and GFAP+ S100β double immunolabelling

Cryocut sections from various groups were processed for double immune fluorescence using simultaneous staining protocol. After blocking with 10% normal goat serum, the sections were incubated with a mixture of rabbit polyclonal anti-GFAP antibody (1:500, DAKO Denmark) and one of the following antibodies: mouse monoclonal A_2_B_5_ (1:200, Abcam) or anti-S100β antibody (1:500, Sigma S2532) overnight at 4°C. Antibody staining was visualized with a cocktail of secondary antibodies, i.e. anti-rabbit FITC conjugated (1:300, Sigma) for GFAP and anti-mouse TRITC conjugated (1:300, Sigma) for A_2_B_5_ and S100β in dark at room temperature for 2 h. All the antibody dilutions were made in 5% BSA in PBS containing 0.5% Tween-20. The sections were finally washed thoroughly with PBS to remove any unbound secondary antibody and cover-slipped with Vectashield Hard+Set mounting medium with DAPI and stored at 4°C, protected from light. The specificity of immunoreactivity was confirmed by omitting the primary or secondary antibody from the procedure.

The images were acquired with the help of Leica DM 6000 Fluorescence microscope using appropriate filters and LAS AF (Leica Application Suite Advanced Fluorescence) imaging software. Identical settings were applied for microscopy and image processing. The relative immunofluorescence intensity of A_2_B_5_, BLBP, GFAP and S100β was quantified using NIH Fiji Image J software. The results were expressed as mean±s.e.m. based on sufficient number of images depending on the available tissue area grabbed from two different sections of three individual HP and LP brains. The total number of images used for quantification at respective time point served as ‘n’ for statistical analysis.

### Statistical analysis

The statistical analysis was performed using Sigma Stat 3.5. Values are expressed as mean±s.e.m. The primary comparison of interest was between HP and LP group at different study time points. All data comparisons were performed using Student's *t*-test between HP and LP group. *P* values less than 0.05 were considered as significant and indicated by asterisk (*) and values less than 0.025-0.001 as highly significant indicated by *** in the graphs.
